# Vitamin C, From Supplement to Treatment: A Re-Emerging Adjunct for Cancer Immunotherapy?

**DOI:** 10.3389/fimmu.2021.765906

**Published:** 2021-11-12

**Authors:** Léonce Kouakanou, Christian Peters, Christine E. Brown, Dieter Kabelitz, Leo D. Wang

**Affiliations:** ^1^ Department of Immuno-Oncology, Beckman Research Institute, City of Hope National Medical Center, Duarte, CA, United States; ^2^ Institute of Immunology, Christian-Albrechts University of Kiel and University Hospital Schleswig-Holstein, Kiel, Germany; ^3^ Department of Hematology and Hematopoietic Cell Transplantation, Beckman Research Institute, City of Hope National Medical Center, Duarte, CA, United States; ^4^ Department of Pediatrics, Beckman Research Institute, City of Hope National Medical Center, Duarte, CA, United States

**Keywords:** immune checkpoint therapy (ICT), CAR (chimeric antigen receptor) T cells, vitamin C (ascorbic acid), cancer immunotherapies, cancer biology

## Abstract

Vitamin C (VitC), in addition to its role as a general antioxidant, has long been considered to possess direct anti-cancer activity at high doses. VitC acts through oxidant and epigenetic mechanisms, which at high doses can exert direct killing of tumor cells *in vitro* and delay tumor growth *in vivo*. Recently, it has also been shown that pharmacologic-dose VitC can contribute to control of tumors by modulating the immune system, and studies have been done interrogating the role of physiologic-dose VitC on novel adoptive cellular therapies (ACTs). In this review, we discuss the effects of VitC on anti-tumor immune cells, as well as the mechanisms underlying those effects. We address important unanswered questions concerning both VitC and ACTs, and outline challenges and opportunities facing the use of VitC in the clinical setting as an adjunct to immune-based anti-cancer therapies.

## Introduction

Vitamin C (or L-ascorbic acid, hereafter referred to as VitC) is a nutrient with a six-carbon structure, synthesized from glucose and abundant in fruits, vegetables and in the kidney and liver of most animals (1). Species such as guinea pigs, fruit bats, and humans are unable to synthesize VitC, due to a mutation in the gene encoding L-gulonolactone oxidase, which catalyzes the last step of VitC synthesis (2, 3). In nature, VitC exists in two different redox forms. The ascorbic acid (reduced) form enters cells using sodium-dependent VitC transporters (SVCTs), whereas the dehydroascorbic acid (oxidized) form enters cells *via* glucose transporters (GLUTs) (4). Inside the cells, the dehydroascorbic acid is reduced back to ascorbic acid which then interacts with different enzymatic systems such as monooxygenases, dioxygenases and hydroxylases, involved in the regulation of numerous biological processes (5). When VitC is not transported inside the cells, it is converted into 2,3-L-diketoglutonate, which is further degraded into oxalate, CO_2_ and L-erythrulose (1). VitC was initially described to play a crucial role in extracellular matrix composition by acting as cofactor for prolyl hydroxylase, the enzyme responsible for collagen biosynthesis (6). Defective collagen synthesis due to VitC deficiency causes scurvy, a bleeding diathesis secondary to poor wound healing.

**Figure 1:**
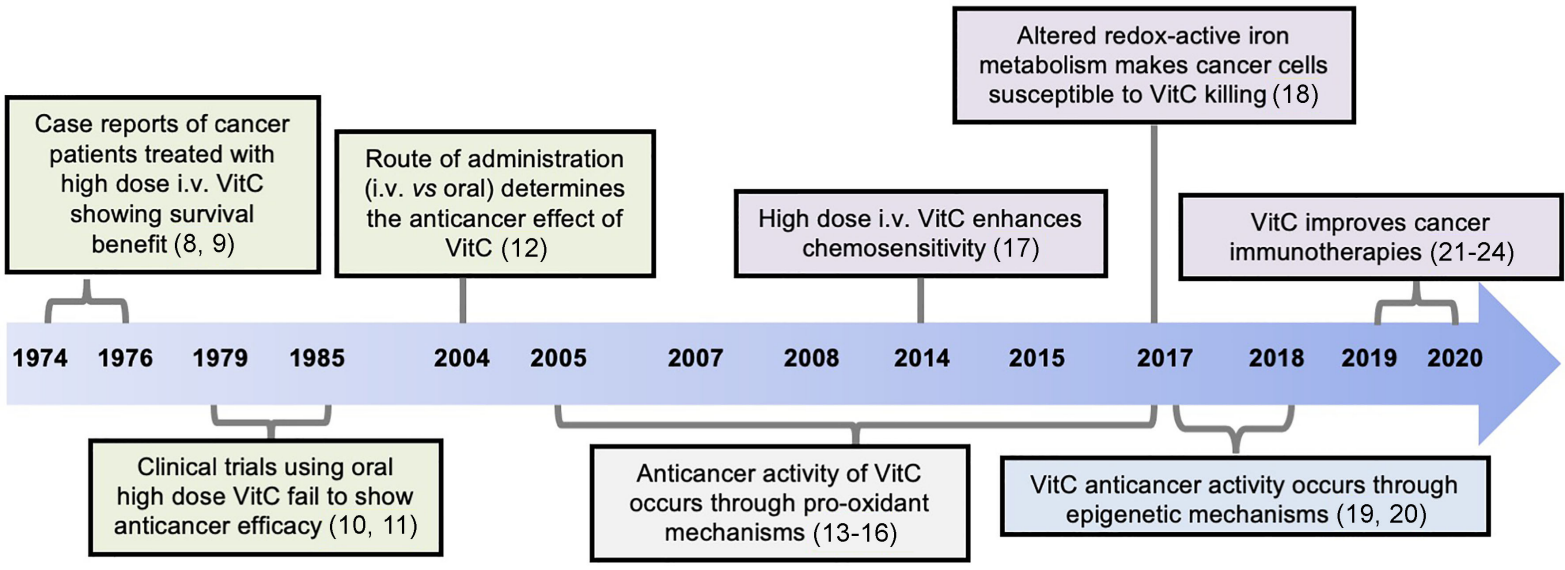
Timeline of discoveries related to the anti-cancer function of Vitamin C (VitC). Advances made over past decades identified VitC as potential anti-cancer agent at high dose yielding remarkable clinical efficacy when given intravenously, and not through oral administration. Mechanistically, VitC at high dose preferentially kills tumor cells *in vitro* or delays tumor growth *in vivo* by exerting pro-oxidant effects and by disrupting iron metabolism, as well as through modulation of epigenetic mechanisms mediated by TET enzymes. Numbers in brackets refer to corresponding references.

VitC has also long been investigated as anti-cancer agent, either in monotherapy or in combination therapy (7), although its effectiveness in cancer has been a subject of controversy. Cameron and colleagues first reported the clinical efficacy of high doses of intravenous (i.v.) VitC in advanced cancer patients (8, 9). In these studies, patients with different types of cancer, who received 10 g of VitC intravenously daily for 10 days and orally thereafter, showed superior overall survival rate compared to the untreated group. These encouraging results could not be reproduced in other early-phase clinical trials, which used the same total dose, delivered entirely orally (10, 11). However, later work revealed that differences in the route of delivery probably explained the discrepant results (12), and it is now proposed that the anti-cancer benefits of VitC require high systemic concentrations that can only be achieved by intravenous delivery. Recent studies have also reported that VitC supplementation at physiologic doses also improves the function of anti-cancer immunotherapies – notably, ACTs- suggesting beneficial roles for VitC at both high (pharmacologic) and low (physiologic) doses. **Figure 1** depicts the timeline in the development of the VitC usage for anti-cancer therapy.

VitC has mostly been understood to exert its anticancer activity through reactive oxygen species-induced oxidative stress (13–18), as well as through modulation of epigenetic programs (19, 20). These mechanisms preferentially affect cancer cells, and VitC is not toxic to normal cells at these doses. Indeed, previous studies have shown a critical and beneficial function of VitC in immune cells, as VitC is present at high intracellular concentrations in lymphocytes (25, 26), and VitC deficiency has been associated with impaired immunity (27). Such immune-modulatory functions of VitC were shown to be regulated at the epigenetic level (28). As it is now quite clear that immune cells have a profound impact on tumors, it is not surprising that recent *in vivo* murine studies have demonstrated that VitC can also contribute to tumor control by modulating the immune system and, interestingly, by enhancing the efficacy of immune checkpoint inhibitor therapy (21–24).

In this review, we focus on what is known about how VitC modulates anti-tumor immune cell function and shed some light on the mechanistic basis of its activity. We also discuss its relevance for current translational immunotherapeutic approaches, highlighting outstanding challenges and unanswered questions as well as current evidence to support the contention that VitC therapy may be a safe and powerful adjunct for cancer immunotherapy, improving efficacy while limiting toxicity.

## Pharmacokinetics of Vitamin C

Following the original observations reporting the controversy on the use of VitC as anticancer agent, pharmacokinetic studies have been performed both in humans and mice to investigate the effects of high-dose VitC, considering different routes of administration and elucidating underlying mechanisms (12, 29). These studies delivered increasing doses of VitC *via* oral and/or i.v. routes and subsequently measured VitC concentrations in the plasma and tissue. Oral administration of high dose VitC resulted in physiologic plasma concentrations, resulting from tight control by factors such as intestinal absorption, tissue transport, and renal reabsorption and excretion (12). In contrast, repeated i.v. administration, which bypasses intestinal regulation, resulted in high plasma and tissue concentrations (12, 29).

## Vitamin C and Tumor Cells

VitC at pharmacologic (high, 1 mM) doses was reported to kill tumor cells *in vitro* and delay tumor growth *in vivo*, essentially through pro-oxidant mechanisms (13, 14). Pharmacologic-dose VitC induces the generation of hydrogen peroxide, which reacts with labile ferrous iron to generate hydroxyl radical known to its action in compromising membrane and DNA integrity as well as glucose metabolism, ultimately leading to cell death (30, 31). Several other mechanisms underlying increased tumor susceptibility to death after high-dose VitC treatment were recently reviewed (32) and include the increased expression of VitC transporters [SVCTs (33) and GLUTs (34)], as well as the decreased concentrations of antioxidant defenses (catalase and superoxide dismutase) and the enhanced cellular levels of prooxidant metal ions (18). These mechanisms likely contribute to VitC-mediated killing of tumor cells; Shenoy et al., testing the effect of VitC on clear cell renal cell carcinoma (ccRCC), showed that short-term exposure (6h) to 1 mM VitC was toxic to ccRCC. However, although the tumor killing effect was forestalled by the addition of catalase, this protective effect disappeared with longer exposures (96h), suggesting an additional, oxidant-independent mechanism of VitC. In subsequent analyses, the authors demonstrated that this effect of VitC was epigenetically mediated (35). Similar observations were also made using lymphoma models (19, 22), and it was shown that pharmacologic-dose VitC exerts antitumor activity through Ten-eleven-translocation (TET)-mediated DNA demethylation (19, 22, 35). In these settings, epigenomic and transcriptomic interrogations from tumor cell samples treated with high-dose VitC revealed enhanced TET-mediated global genome-wide demethylation (increased 5-hydroxymethylcytosine levels) and increased expression of genes encoding human endogenous retroviruses (HERVs) associated with elevated locus-specific demethylation (22). Increased TET expression promotes the effectiveness of cancer immunotherapy (21), and HERVs are known to increase tumor immunogenicity both by increasing tumor mutational burden (36). Furthermore, data from Luchtel et al. showed that VitC-pretreatment of lymphoma cells enhanced their killing by CD8 T cells *in vitro* (22). Together, these data indicate that VitC-facilitated epigenetic modifications enhance tumor immunogenicity, accounting for improved antitumor effect. Based on these and other findings, investigation into high-dose VitC therapy is ongoing in cancer clinical trials.

## Mechanism of action of Vitamin C on Immune Cells

As stated above, VitC acts on tumor cells through oxidant and epigenetic mechanisms. Emerging data indicate that VitC also acts on immune cells in these ways. Free radicals and other reactive oxygen species (ROS), at low dose, are crucial regulators of cell signaling and activation. Indeed, ROS produced in small amounts can positively regulate T-cell receptor (TCR) signaling pathways, thus promoting T-cell activation and proliferation. In support of this, ROS have been shown to be essential for TCR signaling-associated events (37, 38). For example, the moderate generation of ROS following TCR-signaling modulates the phosphorylation of the extracellular signal-regulated kinases (Erk)1/2 (39, 40). In addition, ROS such as H_2_O_2_ can lead to activation of the IκB kinase complex (IKK) (38). However, an overproduction of ROS in the microenvironment causes oxidative stress, leading to damage including cellular dysfunction, cell death, cellular aging, and cancer (41, 42). VitC is a critical non-enzymatic antioxidant that exerts antioxidant activity at micromolar concentrations. This ROS-buffering activity influences cell signal transduction, and the influence of physiologic doses of VitC on TCR signal transduction in general has been recently reviewed. Possible targets include molecules in the proximal TCR signaling complex, as well as downstream signaling molecules such as p38, Erk1/2, NF-κB, NFAT, and PI3K-Akt-mTOR pathway members (43, 44).

Several other studies have described the role of VitC in modulating gene expression in different settings. Duarte et al. reported a genome-wide effect of physiologic-dose VitC in human dermal fibroblasts (45), and Chung et al. identified a number of important genes selectively regulated in human embryonic stem cells cultured in the presence of physiologic dose of VitC (46). Several genes controlling immune function are known to be epigenetically regulated by physiologic dose of VitC, independently of its antioxidant activities. Indeed, compelling evidence suggests that VitC regulates many epigenetic processes, including DNA demethylation and histone modification (28, 46) by interacting with TET (47, 48) and Jumonji C-domain-containing (JmjC) enzymes (49, 50), respectively. TET enzymes convert 5-methylcytosine into 5-hydroxymethylcytosine and further into 5-formylcytosine and 5-carboxylcytosine (47, 48), whereas JmjC demethylases largely regulate chromatin through lysine demethylation of histones (49). VitC-facilitated DNA and histone demethylation is independent of its antioxidant activity. The current model proposes that VitC acts by converting ferric iron (Fe^3+^) into ferrous iron (Fe^2+^), which is essential to maintain the enzymes in their fully catalytic form (51). Interestingly, VitC appears to be highly effective in reducing Fe^3+^ over other reducing agents (52). Several other epigenetic modifying enzymes were also reported to rely on VitC as a cofactor (53), which maintain them in their fully catalytic form, thereby facilitating active gene demethylation crucial for T cell differentiation and function.

Oxidant effects and epigenetic modification are the two best understood mechanisms by which VitC regulates various biological processes. However, the interdependence between these two mechanisms has not yet been investigated. Considering that the availability of VitC inside cells is largely controlled by redox status, Young et al. speculated that redox status in the nucleus could impact the availability of VitC to DNA and histones (28). This view becomes much more complicated by a consideration of the complex and redox-independent influences of other factors, such as cytokines, on epigenetic processes. Indeed, previous murine studies examining the role of IL-2 and IL-6 in the DNA methylation process in regulatory T cells (Treg) have demonstrated the crucial role of IL-2 in the recruitment and binding of TET to the Treg-specific demethylated region (TSDR), whereas IL-6 was reported to hinder that binding (54, 55). Further study is clearly required to fill the gaps in our knowledge of how redox status and epigenetic processes are linked to each other.

## Vitamin C and Antitumor Immune Effects

**Figure 2:**
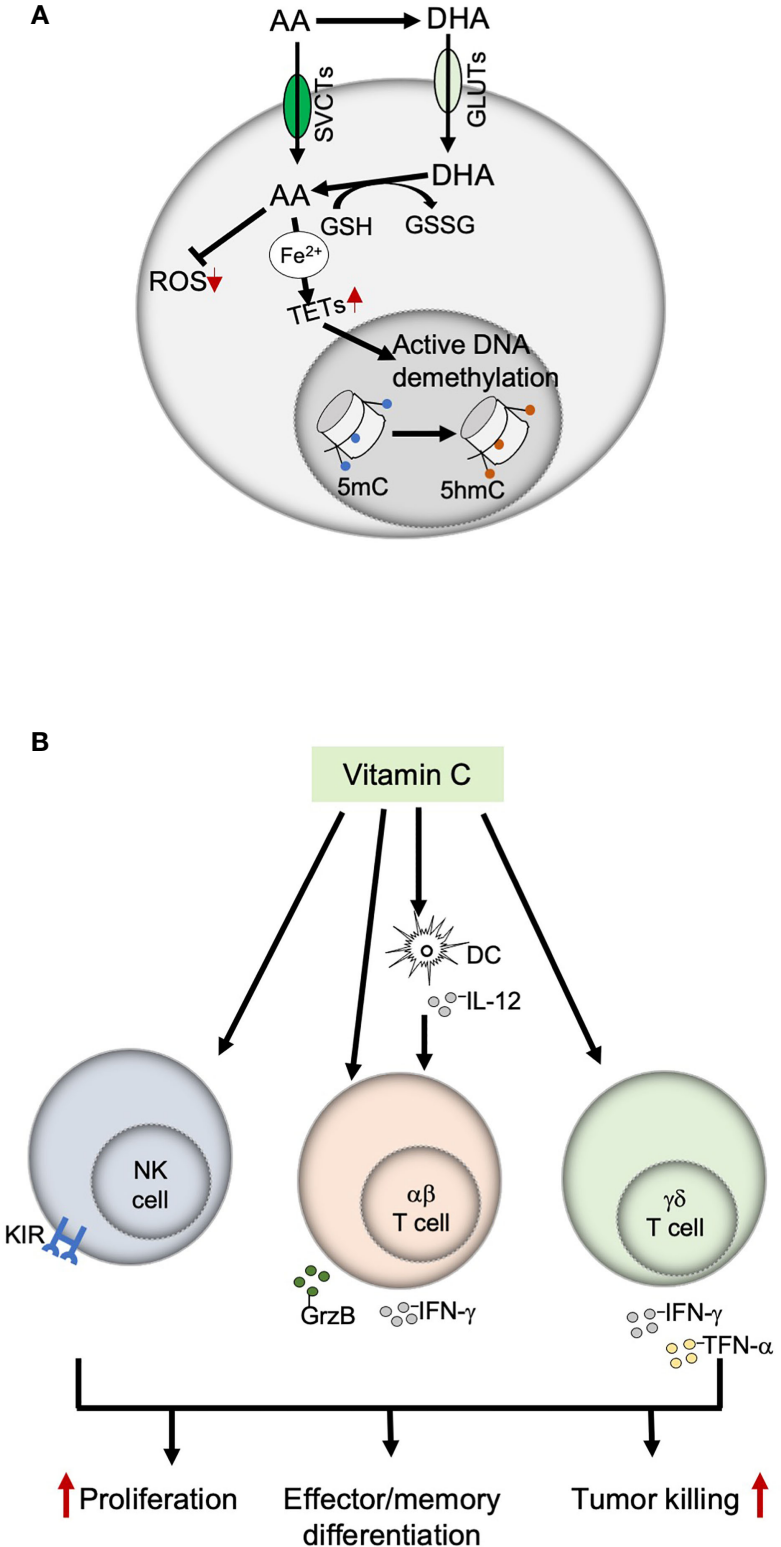
Immunomodulatory functions of Vitamin C. **(A)** Mechanisms of action of Vitamin C; VitC exerts an immune-modulatory effect on immune cells through two main mechanisms, antioxidant activity and epigenetic modulation (by providing ferrous iron to the TET enzymes, which maintains them in their fully catalytic form, thereby ensuring an active DNA demethylation). **(B)** Effects of Vitamin C on immune cells with anti-tumor functions. VitC exerts both direct and indirect effects on NK, αβ and γδ T cells by modulating their proliferation, differentiation, and effector functions. AA, ascorbic acid; DHA, dehydroascorbic acid; GSH, glutathione; GSSG, glutathione disulfide: SVCTs, sodium-dependent vitamin C transporters: GLUTs, glucose transporters: TETs, ten-eleven translocation enzymes; ROS, reactive oxygen species; 5mC, 5-methylcytosine; 5hmC, 5-hydroxymethylcytosine.

In addition to the direct antitumor effect of VitC at high doses, recent studies show that the effect of VitC on immune cells can mediate indirect antitumor effects (22, 23). Interestingly, many of these effects also occur at physiologic doses of VitC. Here, we summarize what is known about the effects of VitC on immune cells with anti-tumor activity (namely NK cells, CD4 and CD8 T cells, and γδ T cells) in the context of cancer immunotherapy. We also discuss the mechanistic basis of its effect with a brief focus on potential targets in TCR signaling. A schematic overview of the influence of VitC on different immune cells is presented in **Figure 2**.

### Natural Killer Cells

NK cells, part of the innate immune system, are capable of rapid and potent killing of virally infected or malignant cells without any prior priming (56, 57). A previous study by Huijskens et al. demonstrated that addition of physiologic-dose VitC to peripheral blood mononuclear cells (PBMC) *in vitro* cultures resulted in a moderately increased proportion of NK cells (58) expressing both inhibitory and activating NK receptors. However, the expression of these receptors was not significantly affected by VitC. Subsequent studies fractionated NK cells further according to their CD56 surface expression to differentiate less mature (CD56^bright^) from more mature (CD56^dim^) NK cells (59–61). Immature CD56^bright^ NK cells exhibit high levels of activating CD94/NKG2C but low frequencies of inhibitory receptors 2DL1 and 2DL3; conversely, mature CD56^dim^ NK cells are 2DL1 and 2DL3 high (59, 60, 62, 63). In this setting, addition of VitC *in vitro* resulted in increased expression of inhibitory receptors on immature CD56^bright^ (both at the gene and protein levels), but not on mature CD56^dim^ NK cells (63). These results suggest that VitC preferentially affects immature NK cells, influencing peripheral NK cell development by inducing a more inhibitory phenotype. It is not known whether VitC influences the developmental expression of other NK cell receptors, or how VitC-mediated changes affect NK cell anti-tumor function. It is possible that VitC-induced upregulation of inhibitory receptors inhibits NK cell cytotoxicity against tumor cells, as upregulation of inhibitory receptors by another epigenetic-modifying drug, decitabine, impaired NK cell anti-tumor activity (64). Furthermore, impaired NK cytotoxic function in the presence of VitC has been reported in *in vitro* studies (58, 65). In contrast, some murine and human (66, 67) studies have reported that VitC augmented the cytotoxic function of NK cells; these studies typically compared VitC depletion with supplementation at physiologic doses, suggesting that there may be an optimal dose range. Clearly, more extensive investigation is needed to fully understand the effect of VitC on NK cell function.

### αβ T Cells

T cells expressing the αβ T-cell receptor (TCR), comprising CD4 and CD8 T cells, release cytolytic granules (68, 69) and produce cytokines, including IFN-γ; after tumor antigen challenge [reviewed in (70)]. Physiologic-dose VitC enhances human T-cell proliferation (44, 71), and exerts both direct and indirect effects on CD4 and CD8 T-cell subsets. A murine study reported that VitC treatment of dendritic cells (DCs) increased phosphorylation of p38, Erk1/2 and NF-κB relevant for DC activation, resulting in elevated production of IL-12, which in turn drove naïve T cells towards the Th1 phenotype, increasing IFN-γ and decreasing IL-5 secretion (72). This was confirmed in a follow-up study demonstrating that murine CD4 and CD8 T cells showed increased IFN-γ production when cocultured with VitC-pretreated DC (73). Furthermore, *in vivo* injection of VitC-pretreated DCs increased IL-12 and IL-15 levels and augmented the generation of memory CD8 T cells; these cells exhibited strong cytotoxic activity against melanoma cells both *in vitro* and *in vivo* (73). Less has been reported on the direct effects of VitC on αβ T cells, and to our knowledge no studies have been reported describing the effects of physiologic-dose VitC on αβ T cells against cancer. Luchtel et al. reported that VitC-pretreatment of isolated human CD8 T cells led to an increase in global 5-hmC levels and enhanced their *in vitro* cytotoxic activity against lymphoma cells (22). Notably, this increase in CD8 T cell cytotoxicity also occurred *in vivo*; however, physiologic-dose VitC has not been tested in this context.

### γδ T Cells

γδ T cells are prototypical unconventional T lymphocytes and express a TCR composed of variable Vδ genes paired with different Vγ elements. Studies have shown that infiltration of γδ T cells into tumors correlates with favorable prognosis in several cancer types (74); however, γδ T cells have not yet been widely adopted as anti-cancer cellular therapies. Approaches to improve γδ T-cell expansion and effector function were recently reviewed (75), and we have previously shown that VitC and its derivative, L-ascorbic acid 2-phosphate (pVC), can increase the *in vitro* proliferation of γδ T cells. We have also shown that pVC and VitC (at low dose) treatment led to reduced intracellular ROS levels, increased proportion of cells in G2/M phase, and increased Ki-67 expression, as well as increased glycolysis and mitochondrial respiration (76). These findings are consistent with the induction of effector and memory programs within γδ T cells (77–79). Indeed, treatment of γδ T cells with physiologic-dose VitC improved *ex vivo* expansion and yielded cell products that expressed higher levels of costimulatory molecules, increased cytokine production, and superior cytotoxic activity against tumor cells (24). These data suggest that VitC may be a useful adjunct for γδ T cell immunotherapies. Along these lines, this study showed that the adoptive transfer of VitC-expanded γδ T cells, but not control γδ T cells, significantly prolonged the survival of humanized mice transplanted with human lung tumor cells (24). Remarkably, a subsequent phase I clinical trial found that repeated infusion of VitC-treated allogeneic γδ T cells increased overall survival rates in lung and liver cancer patients (24).

In summary, VitC appears to exert its effects on immune cells in a dose- and context-dependent manner. At *in vitro* doses above 57 µM, VitC is toxic to human γδ T cells (76); 1mM VitC treatment of human αβ T cells enhanced cytotoxic activity against lymphoma cells (22), but required pretreatment with catalase to protect against VitC-mediated αβ T cell toxicity. Thus, an alternative to the use of pharmacologic-dose VitC may be pVC, which resists oxidation in culture medium and releases the reduced VitC form once inside the cells *via* alkaline phosphatase-mediated hydrolysis (80). pVC therefore has no extracellular prooxidant effect, but still facilitates intracellular biological effects. Indeed, we have seen no toxicity with pVC at doses approaching 1mM, while continuing to see marked metabolic (76) and epigenetic effects (81).

## Vitamin C and Cancer Immunotherapy

### Chimeric Antigen Receptor T Cells

CAR design, biology, and clinical efficacy have been extensively reviewed elsewhere (82, 83). Very briefly, CARs are TCR surrogates with a modular design comprising an antigen-binding domain, an extracellular hinge region, a transmembrane domain, and an intracellular tail incorporating the TCR signaling domain CD3ζ. Despite their overall structural similarity, there are significant differences in proximal signaling after antigen recognition between CARs and TCRs (83, 84). However, given that VitC can modulate activation-induced TCR signaling (43), it is probable that VitC affects proximal CAR signaling. Additionally, γδ T cells treated with pVC have reduced ROS levels, and ROS are known to interact with molecules involved in proximal TCR signaling (85). Moreover, VitC may increase c-Jun levels (43) and Nuclear Factor of Activated T cell (NFAT) activity (86), and both c-Jun and NFAT have been shown to influence CAR T cell function (87). Specific studies on the effects of VitC on CAR signaling have not yet been reported.

Immunomodulatory effects of antioxidants and epigenetic activators on CAR T cell function and development have been described (88–90). Manufacturing anti-CD19-CAR T cells in the presence of the antioxidant N-acetyl-cysteine (NAC) resulted in enforcement of a stem cell memory-like phenotype (T_scm_), including displayed T_scm_-specific metabolic features, improved self-renewal, and superior anti-tumor function *in vivo* (88). Similar observations were also made when manufacturing anti-CD19-CAR T cells in the presence of JQ1, an inhibitor of bromodomain and extra-terminal motif (BET) proteins (90). Interestingly, VitC was also reported to play a crucial role in the generation and maintenance of induced pluripotent stem cells (91), and the combination of NAC and VitC promotes the acquisition of long-term T cell memory in aged mice (92). Additionally, we recently demonstrated that VitC increased the proliferation of IL-2/IL-15-expanded human γδ T cells, which was accompanied by a switch to memory T cell-like metabolism and improved anti-tumor function (24, 76). IL-15-expanded CAR T cells also exhibit an enhanced proliferative capacity and anti-tumor function *in vivo* in part through reduced mammalian target of rapamycin complex 1 (mTORC1) signaling, which enforces a T_scm_ phenotype (93). Taken together, these findings suggest that VitC may be a beneficial addition to the CAR T cell manufacturing process, and we are currently investigating this possibility.

### Immune Checkpoint Therapy

The antagonistic potential of monoclonal antibodies to suppress the function of immune inhibitory receptors such as CTLA-4 and PD-1, known as ICT, has led to remarkable clinical responses against many tumors (94). Unfortunately, ICT is not universally effective (95, 96), and expanding the therapeutic scope of this revolutionary modality would be of great value.

Interestingly, evidence is mounting that VitC can augment the effects of ICT. Recent studies showed that pharmacologic-dose VitC potentiates PD-1 blockade, resulting in increased macrophage and CD8 T-cell tumor infiltration, increased granzyme B production, and significant tumor regression (21, 22). Similarly, addition of high doses of VitC to CTLA-4 and/or PD-1/PD-L1 blockade delayed tumor growth and led to pronounced tumor regression in different tumor mouse models (23). Although mechanistic data are not completely elucidated in these studies, it is interesting that VitC treatment enhances T cell trafficking in solid tumors (97). VitC may also amplify the effects of checkpoint inhibitor therapy through its role as an epigenetic facilitator possibly increasing the expression of retroviral elements and neoantigens (22). Taken together, these studies point the way to therapeutic combination of VitC and ICT in early-phase clinical trials.

## Concluding Remarks

Recent advances in immunotherapy have ushered in a new era in cancer treatment, but many challenges remain to be solved for successful implementation of cancer immunotherapy, including adverse side effects of treatment, off-target toxicity, tumor resistance, tumor evolution, and an immunosuppressive tumor microenvironment, all of which limit translational efficacy across a wide variety of tumors. VitC has recently reemerged as a potent immunomodulatory small molecule, acting on immune cells through well-known antioxidant and epigenetic mechanisms as well as emerging direct signaling effects. Mounting evidence suggests that VitC may be of great therapeutic benefit in combination with immunotherapies, in particular CAR T-cell therapy and immune checkpoint inhibition. Pharmacologic i.v. concentrations of VitC possess anticancer properties, but combinatorial immunotherapeutic approaches may be required for tumor clearance. The addition of physiologic doses of VitC (or pVC) during manufacturing of adoptive cellular therapies may also be beneficial for enhancing T cell proliferation and maintenance of T stem cell phenotype. Although it has been demonstrated that VitC may have some synergistic effects when combined with ACTs, further investigation is needed to better define the optimal dosing, route, and schedule strategies as well as predictive biomarkers of susceptibility of immune/tumor cells to VitC treatment as it pertains to ACT. It is of great interest to study these interactions in more detail, and potentially to incorporate VitC into future immunotherapeutic clinical protocols.

## Author Contributions

LK, LDW, and DK conceived of the manuscript. LK wrote the manuscript and created the figures. LDW, CEB, CP, and DK edited the manuscript and figures.

## Funding

CEB is supported by grants from the California Institute of Regenerative Medicine (CIRM; CLIN2-10248), the National Cancer Institute (NCI, R01CA236500), and the Ivy Foundation. LDW is supported by NCI K08CA201591, CIRM CLIN2-12153, and the Pediatric Cancer Research Foundation. DK was supported by a grant from the Deutsche Forschungsgemeinschaft (Ka 502/19-3).

## Conflict of Interest

CEB receives royalty payments from and is an advisory board member for Mustang Bio.

The remaining authors declare that the research was conducted in the absence of any commercial or financial relationships that could be construed as a potential conflict of interest.

## Publisher’s Note

All claims expressed in this article are solely those of the authors and do not necessarily represent those of their affiliated organizations, or those of the publisher, the editors and the reviewers. Any product that may be evaluated in this article, or claim that may be made by its manufacturer, is not guaranteed or endorsed by the publisher.

## Abbreviations

ACT, adoptive cellular therapy; BET, bromodomain and extra-terminal motif proteins; CAR, chimeric antigen receptor; CTLA-4, Cytotoxic T-lymphocyte-associated protein 4; DC, dendritic cells; Erk, extracellular signal-regulated kinases; GLUTs, glucose transporters; H_2_O_2_, hydrogen peroxide; ICT, Immune checkpoint therapy; IKK, IκB Kinase; i.v., intravenous; mTOR/C1, mammalian target of rapamycin/complex 1; NAC, N-Acetyl-cysteine; NFAT, Nuclear factor of activated T cells; PD-1, Programmed cell death protein 1; pVC, phospho-modified Vitamin C; ROS, reactive oxygen species; SVCTs, sodium-dependent VitC transporters; TCR, T-cell receptor; TET, ten-eleven-translocation; VitC, Vitamin C.
